# Integrated transcriptomics and metabolomics analysis of catechins, caffeine and theanine biosynthesis in tea plant (*Camellia sinensis*) over the course of seasons

**DOI:** 10.1186/s12870-020-02443-y

**Published:** 2020-06-29

**Authors:** An-Dong Gong, Shuai-Bin Lian, Nan-Nan Wu, Yong-Jie Zhou, Shi-Qi Zhao, Li-Min Zhang, Lin Cheng, Hong-Yu Yuan

**Affiliations:** 1 Henan Key Laboratory of Tea Plant Biology, College of Life Science, Xinyang Normal University, Xinyang, 464000 People’s Republic of China; 2 College of Physics and Electronic Engineering, Xinyang Normal University, Xinyang, 464000 People’s Republic of China; 3 CAS Key Laboratory of Magnetic Resonance in Biological Systems, State Key Laboratory of Magnetic Resonance and Atomic and Molecular Physics, National Centre for Magnetic Resonance in Wuhan, Wuhan Institute of Physics and Mathematics, Chinese Academy of Sciences (CAS), Wuhan National Research Center for Optoelectronics, Wuhan, 430071 China

**Keywords:** Transcriptomics, Metabolomics, Catechins, Caffeine, Theanine, Seasons

## Abstract

**Background:**

Catechins, caffeine, and theanine as three important metabolites in the tea leaves play essential roles in the formation of specific taste and shows potential health benefits to humans. However, the knowledge on the dynamic changes of these metabolites content over seasons, as well as the candidate regulatory factors, remains largely undetermined.

**Results:**

An integrated transcriptomic and metabolomic approach was used to analyze the dynamic changes of three mainly metabolites including catechins, caffeine, and theanine, and to explore the potential influencing factors associated with these dynamic changes over the course of seasons. We found that the catechins abundance was higher in Summer than that in Spring and Autumn, and the theanine abundance was significantly higher in Spring than that in Summer and Autumn, whereas caffeine exhibited no significant changes over three seasons. Transcriptomics analysis suggested that genes in photosynthesis pathway were significantly down-regulated which might in linkage to the formation of different phenotypes and metabolites content in the tea leaves of varied seasons. Fifty-six copies of nine genes in catechins biosynthesis, 30 copies of 10 genes in caffeine biosynthesis, and 12 copies of six genes in theanine biosynthesis were detected. The correlative analysis further presented that eight genes can be regulated by transcription factors, and highly correlated with the changes of metabolites abundance in tea-leaves.

**Conclusion:**

Sunshine intensity as a key factor can affect photosynthesis of tea plants, further affect the expression of major Transcription factors (TFs) and structural genes in, and finally resulted in the various amounts of catechins, caffeine and theaine in tea-leaves over three seasons. These findings provide new insights into abundance and influencing factors of metabolites of tea in different seasons, and further our understanding in the formation of flavor, nutrition and medicinal function.

## Background

The tea produced from *Camellia sinensis* is an important non-alcoholic beverage that was daily consumed by more than 3 billion people of 160 countries worldwide [1]. Currently, it is reported that tea plants are commercially cultivated in over 100 countries, and more than 5 million tons of tea beverages were produced every year [2–4]. The story of tea starts in China in 2737 BC. China remians the largest tea producer with an output of 1.9 million tons annually [5, 6] with more than 100 officially approved tea cultivars in 15 provinces [7, 8]. Xinyang maojian tea is one of the Chinese traditional famous teas with a specific flavor. Some evidence indicates that these specific flavors are highly related to the abundance of specific secondary metabolites such as polyphenols, theanine, caffeine, vitamins, volatile oils, polysaccharides, and minerals [2, 3, 6]. Among these metabolites, polyphenols, caffeine, and theanine are usually considered as distinctive functional compounds, which play key roles in the formation of special taste, as well as nutritional and medicinal properties [6, 9–11].

As one of the distinctive polyphenols, catechins contribute 12–24% of dry weight to tea leaves [8] and estimate 70–75% of the bitterness and astringency of green tea [12]. Catechins in tea-leaves includes four free types including (+)-catechin (C), (−)-epicatechin (EC), (+)-gallocatechin (GC), (−)-epigallocatechin (EGC), and four gallate types including (−)-catechin-3-gallate (CG), (−)-epicatechin-3-gallate (ECG), (−)-gallocatechin-3-gallate (GCG), (−)-epigallocatechin-3-gallate (EGCG). Of them, gallate catechins are major contributers to the bitterness and astringency [13]. Moreover, clinical studies have shown the beneficial effects of catechins on humans such as antioxidant activities on cardiovascular health [14], inhibiting of cancer cells growth, inhibiting of blood clots formation, reduction of platelet aggregation and lipid regulation [15].

Theanine as another important compound in tea leaves is a non-protein amino acid and contributes 1–2% to the dry weight of leaves [16] which endows the sweet and savory taste to some tea beverages [11]. Theanine is originally biosynthesized in tea roots, then transfers to the growing shoot through the phloem, and finally accumulates in the developing leaves. Several studies have revealed that the biosynthesis of theanine in the tea plants was highly related to environmental factors such as sunlight and heat [11]. Theanine can be hydrolyzed back to ethylamine, and further utilized as precursors in catechins biosynthesis in long-term sunlight exposure. On the other hand, tea plants growing in reduced sunlight exposure present a higher concentration of theanine and lower amounts of catechins. Additionally, the theanine also shows potential health benefits in humans, such as enhancingement relaxation and immune system, improving concentration and learning ability, prevention of certain cancers, promoting weight loss [11].

Caffeine as an important member of methylxanthine contributes 3% to the dry weight of tea leaves, which is always considered as a marker to evaluate the tender of tea leaves [17]. In tea beverage, caffeine has a bitter taste and significantly contributes to the briskness of tea [16]. Caffeine can also stimulate the central nervous system and enhance mental and physical processes in the human body [18]. The Food Standards Agency strongly recommends that pregnant women should drink caffeine less than 300 mg every day due to its potential risk of spontaneous miscarriage or low birth weight of infants [19].

It has been showed that the amounts of catechins, caffeine, and theanine are determined by factors such as tea varieties [6], altitudes [20], rolling methods and processing stages [16]. However, the information concerning the season changes of these compounds is underestimated. Therefore, an integrated transcriptomics and metabolomics method was used in this study to quantitatively investigate the dynamic changes of these compounds in tea-leaves of different seasons, and to elucidate the correlated major genes and transcriptional factors relating to these amount changes.

## Results

### Phenotypic characteristics of tea samples

**Fig. 1:**
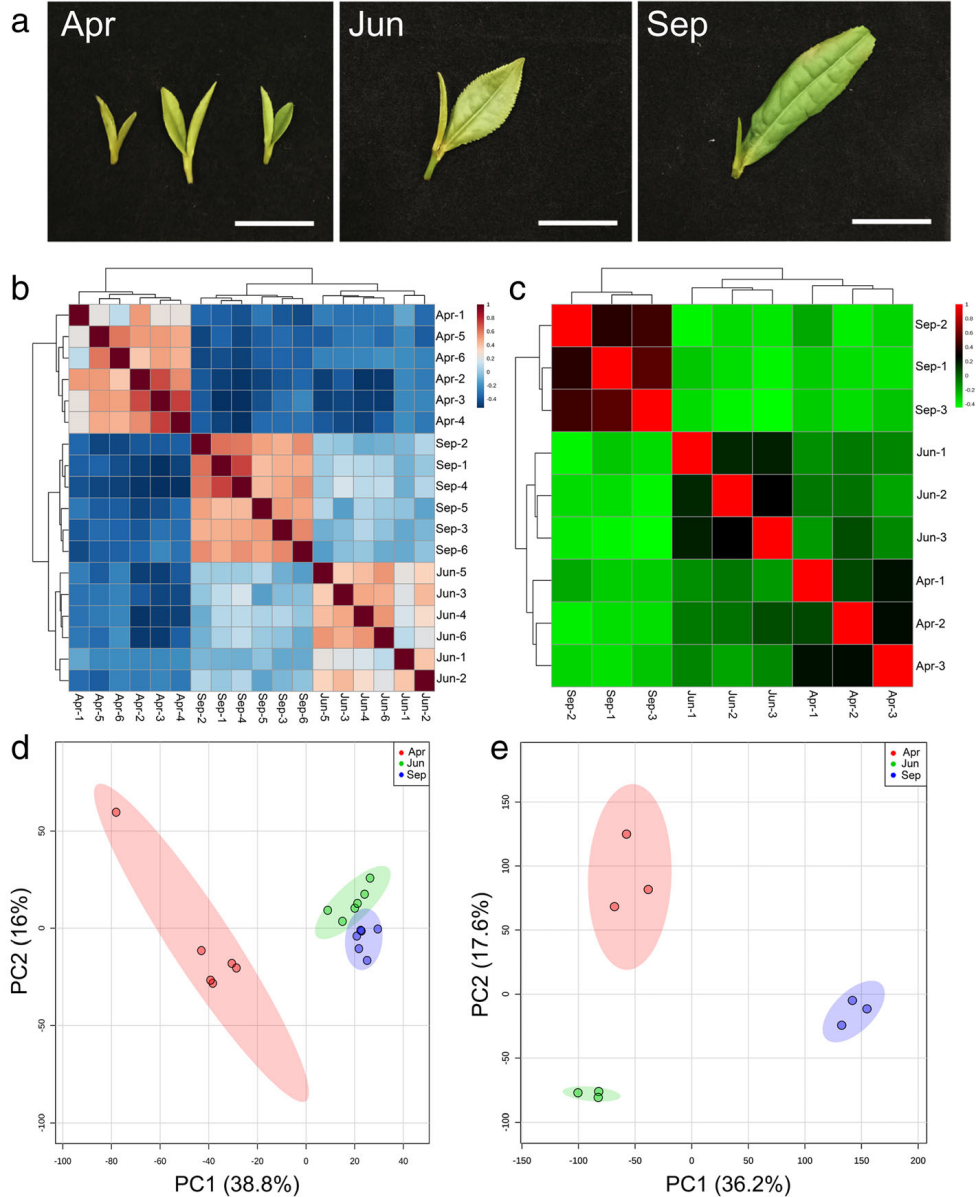
Phenotype analysis and systematic analysis of transcriptome and metabolome data of tea samples in different seasons. **a** Phenotype of tea samples collected from different season, **b c**. Correlation heatmaps of data in metabolome and transcriptome respectively. **d**, **e**. Principal component analysis of data obtained in metabolome and transcriptome respectively. The explained variances are shown in brackets. Spr: Spring; Sum: Summer; Aut: Autumn. Bar = 15 mm

The obtained tea tissues in three seasons (Spring, Summer, and Autumn) showed varied phenotypes. The bud and first leaf were tender in Spring (April) than the samples collected in Summer and Autumn. Color of tea tissues was light green, green and light yellow in Spring, Summer, and Autumn, respectively. Additionally, the length of first leaves and buds, and the ratio of leave to bud in each collected sample were calculated. The results showed that the length ratio in autumn was 4, which was higher than that in Summer (1.6) and Spring (0.8) (Fig. 1A).

### Metabolic and genetic divergence

The metabolic profiling of tea samples in different seasons (6 replications in each) were obtained through an untargeted ultra-high-performance liquid chromatography/quadrupole time-of-flight mass spectrometry (UPLC/Q–TOF–MS/MS) system. Of predominantly detected 4840 peaks, 3083 peaks were identified by searching against the Human Metabolome Database (HMDB) and tea metabolome database (http://pcsb.ahau.edu.cn:8080/TCDB/f). The low quality data (empty data in each group over 60%, 270 compounds) were removed from those of identified compounds in each sample. After filtration, 2813 identified putative peaks were used for downstream statistical analysis.

Heatmap analysis of metabolomics data showed that 18 samples were significantly separated into three clusters corresponding to three seasons (Spring, Summer, and Autumn) (Fig. 1b), indicating that the tea samples collected from three seasons exhibited different metabolic characters. Similarly, transcriptomics data were also clearly clustered into three groups suggesting different genetic characters of tea samples from three seasons (Fig. 1c). Consistently, three distinct groups were identified based on both Principal Component Analysises (PCAs) of metabolomics and transcriptomics datasets(Fig. 1a and e).

### Identification, classification, and verification of differentially expressed gene (DEG)

**Fig. 2:**
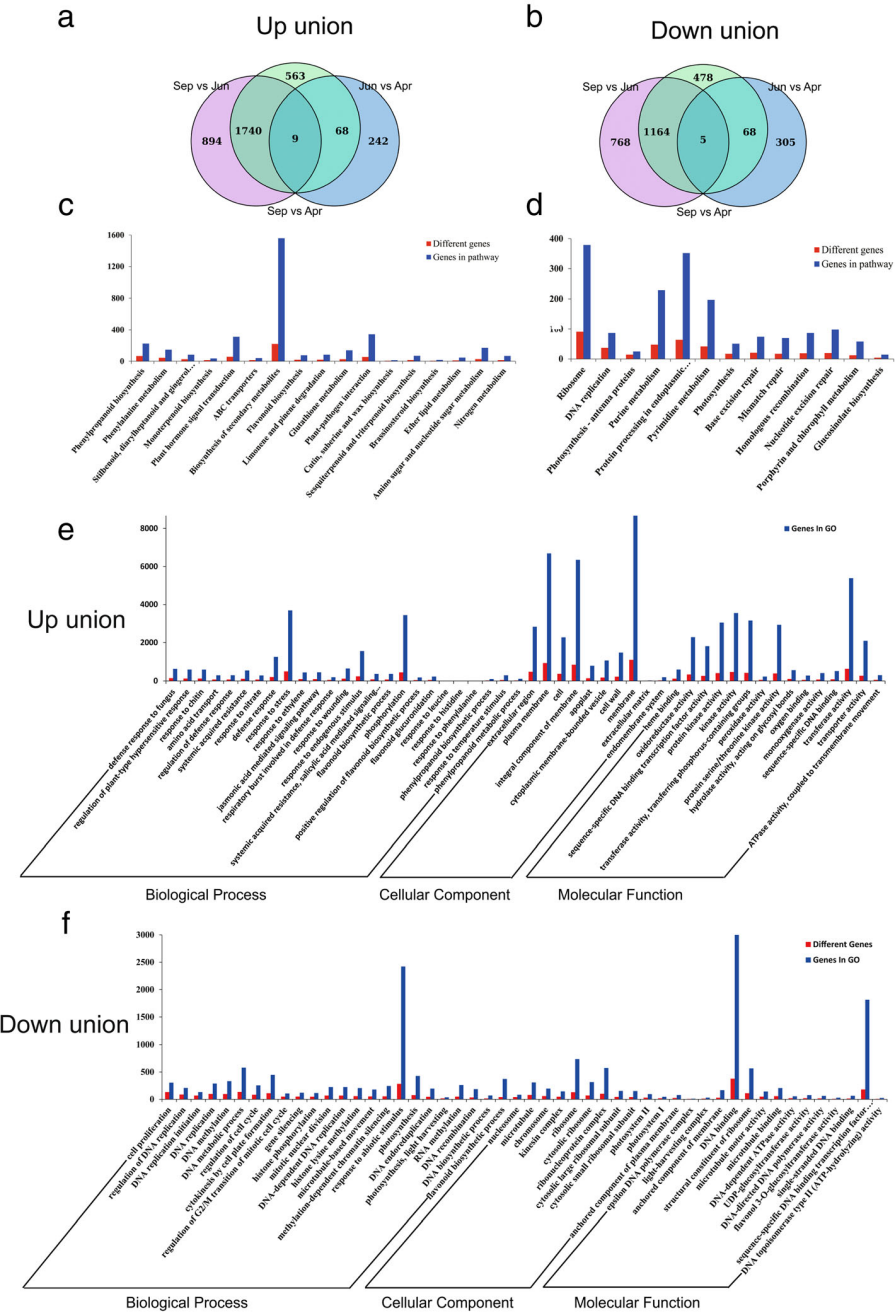
Statistical analysis of up and down regulated genes of in transcriptome. The pairwise comparisons between three samples were conducted including Summer vs Spring, Autumn vs Summer, Autumn vs Spring. All up (down) regulated genes with significant differences were collected and analyze in GO and KEGG database. **a**, **b**. Venn diagram analysis of up and down regulated union gene, respectively. **c**, **d** KEGG enrichment analysis of up and down regulated union genes, respectively. **e**, **f** Go enrichment analysis of up and down regulated genes, respectively. Spr: Spring; Sum: Summer; Aut: Autumn

More than 6 Gb raw data were obtained from the transcriptome resulting in 41.8 to 49.0 million total reads for each sample. 82.23, 77.83 and 81.20% of clean reads were concordantly mapped to the tea genome sequence, for the samples from April, June and September, respectively (Table S1). Additionally, 3516 up-regulated and 2788 down-regulated genes were obtained from a pair-wise comparative analysis under the criteria of |log2FC| > 1 and FDR < 0.05 (Table S2). For the tea samples collected in the different seasons, 894, 242 and 563 up-regulated DEGs were overlapped in the comparsions of September vs June, September vs April, and June vs April (Fig. 2a). 768, 305, and 478 down-regulated genes were also detected and overlapped in the comparisons of September vs June, September vs April and June vs April, respectively (Fig. 2b). Moreover, nine up-regulated and five down-regulated genes were found in all three seasonal comparisons (Fig. 2a, b).

Pathway analysis was subsequently conducted based on the Kyoto Encyclopedia of Genes and Genomes (KEGG) database (Table S3). Up-regulated genes are mainly grouped under the terms of phenylpropanoid biosynthesis, phenylalanine metabolism, stilbenoid diarylheptanoid, and gingerol biosynthesis. Other terms related to catechins biosynthesis including flavonoid and secondary metabolites biosynthesis were also found (Fig. 2c). Down-regulated genes were significantly enriched in the 13 KEGG terms, and most of these terms are related to photosynthesis and nucleic acid metabolism including ribosome, DNA replication, photosynthesis-antenna proteins, photosynthesis, base excision repair, mismatch repair, homologous recombination, and nucleotide excision repair (Fig. 2a).

Genes in Gene Ontology (GO) terms could be divided into three dominant categories: biological process (BP), molecular function (MF) and cellular component (CC) category (Table S4). In the BP category, the up-regulated genes were significantly enriched in response to stress, and phosphorylation terms, and the top three enriched terms are defense response to fungus, protein targeting to membrane, regulation of plant-type hypersensitive response (Fig. 2e). The terms related to flavonoid biosynthetic process, including flavonoid biosynthetic process, positive regulation of flavonoid biosynthetic process, flavonoid glucuronidation, response to phenylalanine, phenylpropanoid biosynthetic process, response to temperature stimulus and phenylpropanoid metabolic process, were also denriched (Fig. 2e). The down-regulated genes were significantly enriched in response to abiotic stimulus terms, and the top associated six terms are related to DNA replication and cell proliferation, regulation of DNA replication, DNA replication initiation, DNA replication, DNA methylation, and DNA metabolic process. Other terms related to DNA biosynthesis were also observed (Fig. 2f). In the CC category, the primary three GO terms for up-regulated genes are extracellular regions including plasma, membrane, and cell (Fig. 2e). Some other terms related to cell components, including integral components of membrane, apoplast, cell wall and membrane, and endomembrane system, were observed (Fig. 2e). The primary three terms for down-regulated genes are nucleosome, microtubule, and chromosome, which are related to nucleic acid (Fig. 2f). In the MF category, for up-regulated genes, the top three terms are heme binding, oxidoreductase activity, and sequence-specific DNA binding transcription factor activity (Fig. 2e). The top three terms for down-regulated genes are DNA binding, structural constituent of ribosome, and microtubule motor activity (Fig. 2f).

**Fig. 3:**
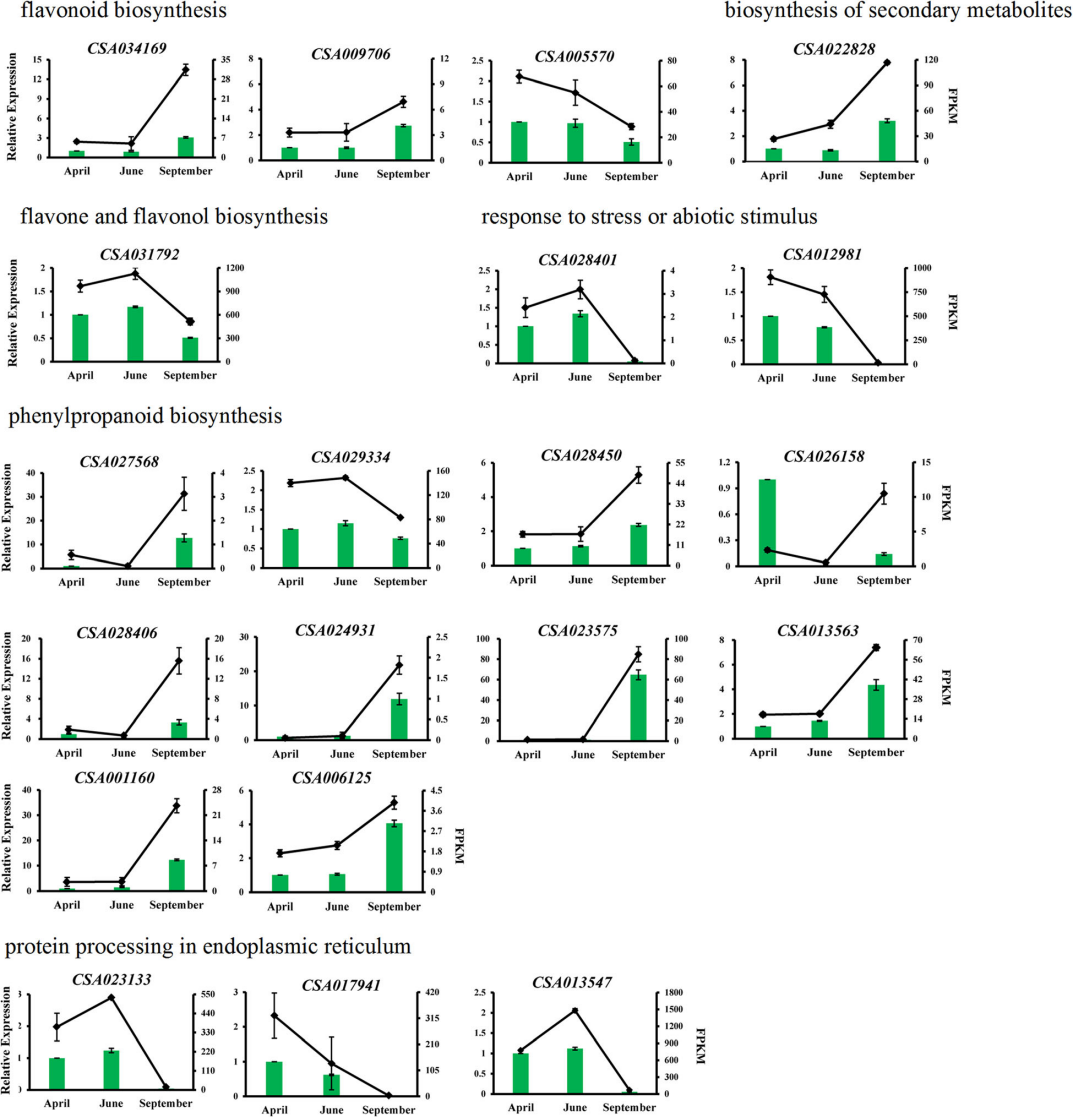
Validation of the relative expression levels of DEGs by qRT-PCR. Histogram graph means the data in transcriptome analysis, and line indicates the data of qRT-PCR

The reliability of the RNA-Seq data was verified by a quantitative real-time polymerase chain reaction (qRT-PCR). Twenty DEGs were randomly selected for the qRT-PCR test, and these DEGs were predicted to be related with flavonoid biosynthesis (CSA034169, CSA009706, CSA005570), flavone and flavonol biosynthesis (CSA031792), phenylpropanoid biosynthesis (CSA029334, CSA028450, CSA028406, CSA027568, CSA026158, CSA024931, CSA023575, CSA013563, CSA001160, CSA006215), biosynthesis of secondary metabolites (CSA022828) and protein processing in endoplasmic reticulum (CSA023133, CSA017941, CSA013547), response to stress or abiotic stimulus (CSA028401, CSA012981). Of selected 20 genes, the expression of one gene (CSA026158) was not agreed with the RNA-Seq data, other 19 genes (95% of 20 genes) were coherent with the RNA-Seq data. The observed expression patterns of the genes of interest were consistent with previous the RNA-Seq data confirmed the reliability of the RNA-Seq results (Fig. 3).

### Targeted quantification of catechins, caffeine, and theanine

**Fig. 4:**
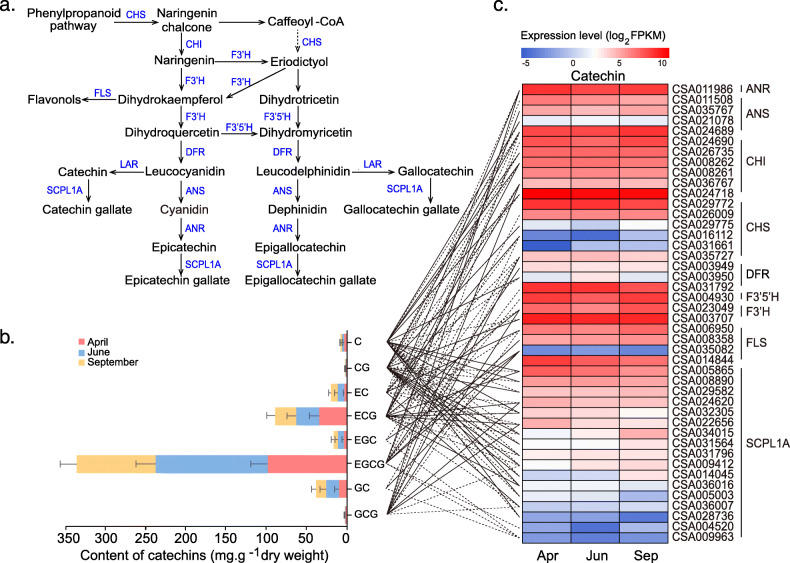
Gene expression, catechins content and the correlations between them in three different seasons. **a**. The biosynthesis pathway of catechins in tea plants. **b**. The concentration of each catechin in tea leaves of three seasons. **c** Heat map of detected genes in catechins biosynthesis pathway of transcriptome data. The correlation between **b** and **c** were indicated with straight lines (coefficients> 0.9, *P* < 0.05). Full line means positive correlation, dotted line means negative correlation. ANR: anthocyanidin reductase; ANS: anthocyanidin synthase; CHS: chalcone synthase; CHI: chalcone isomerase; DFR: dihydroflavonol 4-reductase; F3’H: flavanone 3-hydroxylase; F3’5’H: flavonoid 3′,5′-hydroxylase; SCPL1A: type 1A serine carboxypeptidase-like acyltransferases

**Fig. 5:**
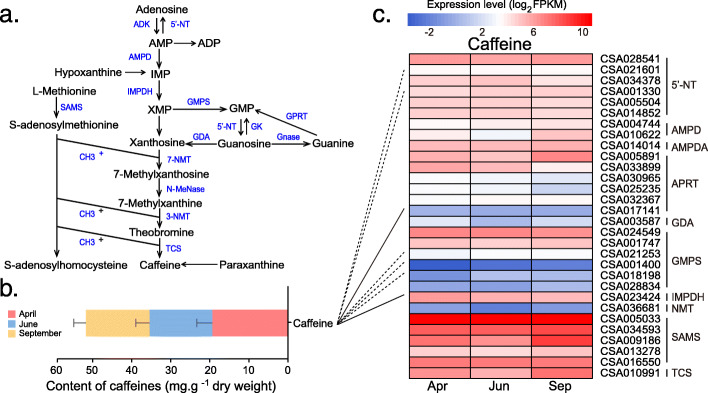
Gene expression, caffeine content and the correlations between them in three different seasons. **a**. The biosynthesis pathway of caffeine in tea plants. **b** The concentration of caffeine in tea leaves of three seasons. **c** Heat map of detected genes in caffeine biosynthesis pathway of transcriptome data. The correlation between **b** and **c** were indicated with straight lines (coefficients> 0.9, P < 0.05). Full line means positive correlation, dotted line indicates negative correlation. 5′-NT: 5′-nucleotidase; AMPD: AMP deaminase; APRT: adenine phosphoribosyltransferase; GDA: guanine deaminase; GMPS: GMP synthase; IMPDH: IMP dehydrogenase; NMT: N-methyltransferase; SAMS: S-adenosyl-L-methionine; TCS: caffeine synthase

**Fig. 6:**
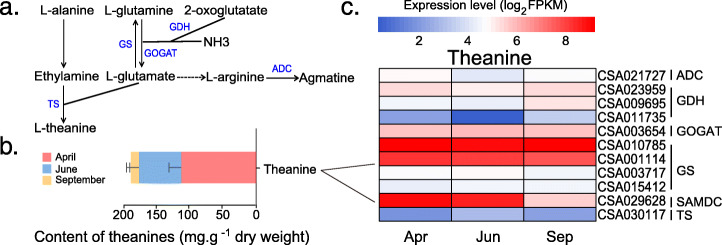
Gene expression, theanine content and the correlations between them in three different seasons. **a**. The biosynthesis pathway of theanine in tea plants. **b** The concentration of theanine in tea leaves of three seasons. **c** Heat map of detected genes in theanine biosynthesis pathway of transcriptome data. The correlation between **b** and **c** were indicated with straight lines (coefficients> 0.9, *P* < 0.05). Full line means positive correlation, dotted line indicates negative correlation. TS: theanine synthetase; GS: glutamine synthetase; GOGAT: glutamate synthase; GDH: glutamate dehydrogenase; SAMDC: S-adenosylmethionine decarboxylase; ADC: arginine decarboxylase

Quantitative analysis of catechins, caffeine, and theanine in the tea samples obtained from three seasons were performed by HPLC method (Fig. 4b, 5Bband 6b). The metabolite (caffeine, catechins, and theanine) content also showed dynamic changes over three seasons. The total content of catechins in tea leaves of June (Summer) (203.44 mg/g) was higher than that in April (Spring) (151.88 mg/g) and September (Autumn) (153.19 mg/g). Eight kinds of catechins including C, EC, EGC, GC, CG, ECG, EGCG, and GCG were detected in the tea leaves. Among these catechins, EGCG abundance was always higher than other compounds in any of tea samples. Furthermore, EGCG abundance in June (139.60 mg/g) was higher than that in April (98.22 mg/g) and September (97.97 mg/g). ECG was the second abundant compound ranged from 26.07 to 33.85 mg/g in different seasons (Fig. 4b). No significant difference was observed in caffeine abundance for the tea leaves obtained from different seasons (Fig. 5b).Besides, the theanine abundance in the tea leaves collected in April was 113.64 mg/g, which was higher than that in June (63.98 mg/g) and September (11.68 mg/g), respectively (Fig. 6b).

### Correlation analysis between gene expression and metabolites abundance

Transcriptomics data showed that 14 genes were involved in the catechins biosynthetic pathway. Catechins biosynthesis was derived from phenylpropanoid, regulated by more than 10 genes, and synthesized into four kinds of catechins (named as C, EC, EGC, and GC), which were further catalyzed by SCPL1A (type 1A serine carboxypeptidase-like acyltransferases) into gallate catechins (named as CG, ECG, EGCG, and GCG) (Fig. 4a). In transcriptome data, we detected nine genes that are involved in catechins biosynthesis, which are chalcone synthase (CHS, 6 copies), chalcone isomerase (CHI, 6 copies), anthocyanidin reductase (ANR), F3’H (flavanone 3-hydroxylase, 2 copies), F3′5′H (flavonoid 3′,5′-hydroxylase), DFR (dihydroflavonol 4-reductase, 2 copies), FLS (flavonol synthase, 4 copies), ANS (anthocyanidin synthase, 4 copies), and LCR (leucoanthocyanidin reductase). Some of these genes with multicopies showed different expression levels in Spring, Summer, and Autumn. Additionally, the expression of these genes was highly correlated with the concentration of the catechins in three seasons (Pearson’s correlation test> 0.9, *p* < 0.05) (Table S5).

Within the caffeine biosynthetic process, adenosine, a basic substrate, was catalyzed by about 10 enzymes and enventually formed as caffeine through more than 10 steps (Fig. 5a). The correlation analysis showed that 5′-NT (5′-nucleotidase) and GMPS (GMP synthase), genes were negatively correlated with caffeine abundance; APRT (adenine phosphoribosyltransferase), and IMPDH (IMP dehydrogenase), genes were positively correlated with caffeine abundance in tea leaves (Fig. 5c). L-theanine was de novo synthesized from some substrates such as L-alanine, L-glutamine, and 2-oxoglutarate. The biosynthesis of caffeine were mediated by five important genes including TS (theanine synthetase), GS (glutamine synthetase), GOGAT (glutamate synthase), GDH (glutamate dehydrogenase), SAMDC (S-adenosylmethionine decarboxylase), and ADC (arginine decarboxylase) (Fig. 6a). ADC, GOGAT, SAMDC and TS genes were single copy, whileGDH and GS genes were multiple copies. The correlation analysis showed that GS and SAMDC genes were positively correlated with theanine abundance in tea leaves (Fig. 6c).

### Identification of the transcription factors relating to the mainly metabolites content changes

**Fig. 7:**
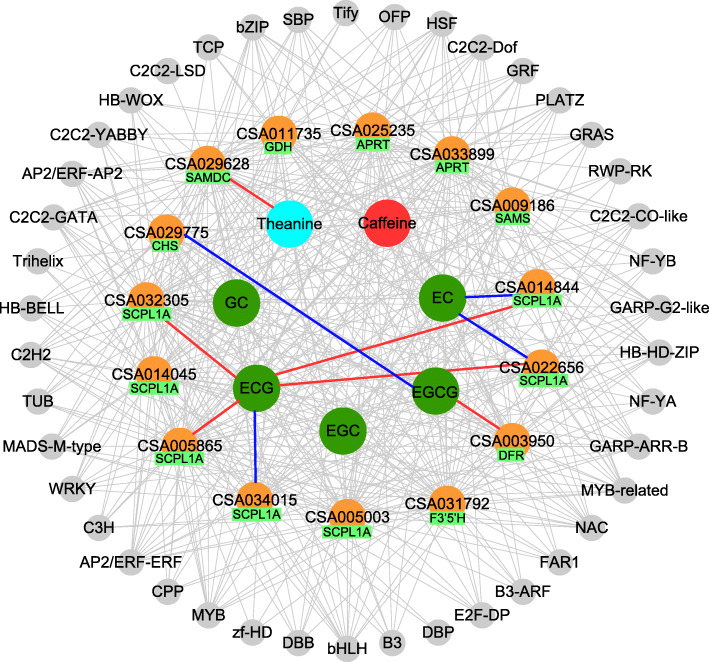
Transcription factor regulation networks on catechins, caffeine, theanine gene expression and metabolite biosynthesis. The genes related with the expression of TFs were listed in the middle cycle, and red line means positive correlation, blue line means negative correlation between compound content and gene expression. EC: (−)-epicatechin; GC: (+)-gallocatechin; EGC: (−)-epigallocatechin; EGCG: (−)-epigallocatechin-3-gallate; ECG: (−)-epicatechin-3-gallate

Transcription factors (TFs)-gene expression-metabolites networks were constructed to identify the important TFs potentially associated with the mainly metabolites content changes (Fig. 7, Table S6). Totally, 76 TFs belonging to 42 TF families were included in the network. TFs with multiple replications were categorized into NAC (6 TFs), AP2/ERF-ERF (4 TFs), WRKY (4 TFs), and bHLH (3 TFs) families. These TFs showed a highly correlation with the expression of genes catechins (CHS, F3’5’H, SCPL and DFR), caffeine (APRT and SAMS) and theanine (SAMDC and GDH genes) biosynthesis with weight > 0.5. Some of these enriched genes highly correlated with metabolites abundance changed dramatically in three seasons. In the catechins biosynthetic process, EGCG, the most abundant catechin, was positively correlated with the expression of the DFR gene (CSA003950) and negatively correlated with the CHS gene (CSA029775). ECG was positively correlated with four SCPL1A genes (CSA032305, CSA005865, CSA022656, CSA014844), and negatively correlated with one another SCPL gene (CSA034015). For the theanine biosynthesis, the SAMDC gene (CSA029628) was found to be highly correlated with TFs and positively correlated with theanine abundance in tea leaves of different seasons. However, caffeine biosynthesis exhibited less changes than other two compounds among different seasons. Four genes (CSA011735, CAS025235, CSA033899, CSA009186) were correlated with TFs, which showed no significant correlation with caffeine abundance of tea-leave obtained from different seasons (Fig. 7).

## Discussion

The content of the catechins, caffeine, and theanine, three important compounds, mainly contributed to flavor, nutrition and medicinal properties in the tea leaves, vary from season to season. However, the dynamic changes of these compounds in tea leaves, as well as the potential affected factors are still undetermined. Here, an integratation of transcriptomic and metabolomic approach was used to analyze the candidate factors affecting the abundance changes of metabolites, and to further elucidate the correlations among metabolites abundance, gene expression and TFs of fresh tea leaves obtained from Spring (April), Summer (June) and Autumn (September).

It is conceivable that the tea growing in different seasons exhibits various phenotypes. For instance, it shows lighter green color and lower leave/bud ratio in Spring than those in Summer and Autumn (Fig. 1), which may attributed to the varied cultured enviroments, such as varied temperature, humidity, and sunshine intensityAdditionally, we further proved that the content of catechins in Summer is higher than that in Spring and Autumn (Fig. 4b), whereas, the theanine content is higher in the Spring than that in the Summer and Autumn (Fig. 6b). Likewise, Chen et al. [20] also proved that the content of catechins not only show differences in varied seasons, they were also varied in the same mountains of different altitudes. The concentration of catechins in fresh tea leaves was inversely correlated to the cultivation altitude. Similar results have been proved in the previous work that EGCG as the most abundant catechins in tea leaves exhibited higher levels in Summer than that in Spring [21, 22].. So far, numerous researches have proved that sunlight plays key roles in the biosynthesis of tea metabolites [23]. Longer daylight hours in Summer with more efficient photosynthesis enriched more catechins than that in Spring and Autumn [22]. Similarly, theanine biosynthesis in tea plant was regulated and controlled by sunshine duration [11]. It can be transformed into catechins in tea shoots under long sunshine exposure. Therefore, more abundant theanine was observed in Spring and Autumn [11]. However, caffeine abundance in tea leaves of different seasons showed less change probably due to no significant effect of photosynthesis activity on caffeine biosynthesis. In the current analysis, we found that most DEG enriched in the KEGG pathway related to metabolite biosynthesis (phenylpropanoid, monoterpenoid, secondary metabolite, flavonoid, sesquiterpenoid and triterpenoid, cutin, suberin, and wax) were up-regulated. Whereas, the genes enriched in photosynthesis and photosynthesis-antenna proteins were down-regulated. These evidence together with quantification of three metabolites revealed that photosynthesis may play key roles in the regulation metabolite production, as well as the formation of different tea characters in line with the seasons’ change.

In the process of tea plant growth and development, tea genome is the product of two rounds of whole-genome duplications which resulted in abundant copies of important genes in the biosynthesis of secondary metabolites [8]. Namely, these genes may directly or indirectly play key roles in the regulation of metabolites abundance in tea leaves. Furthermore, transcription factors (TFs) also mediates the genes expression by binding to DNA to affect the activity of the enzyme which may further regulate the production of metabolite in tea plants [24–27]. The correlation analysis between structural genes and TFs provides effective approaches for identifying important TFs [27]. Till now, many studies demonstrated that TFs were involved in the regulation of catechins (MYB, bHLH, MADS, R2R3-MYB families) [28], caffeine (bZIP, bHLH, GATA and MYB families), and theanine biosynthesis (AP2-EREBP, bHLH, C2H2, and WRKY families) [29]. Most of these TFs were also detected in our work and proved valid in the regulation of 15 structural genes in the biosynthesis of catechin (CHS, SCPL1A, F3’5’H, DFR), caffeine (APRT, SAMS) and theatine (SAMDC and GDH) (Fig. 7). Whereas, the interactions among TFs, structural genes and metabolite biosynthesis are complex in the tea plants, there are thus many other important TFs in regulation catechins, caffeine, and theanine biosynthesis are still unreported. In current work, apart from these traditional TFs mentioned before, some other TFs from TCP, SBP, CPP, GRF, FAR1 families were also found correlated with the biosynthesis of catechins, caffeine and theanine. However, due to a small number of samples used, some identified TFs might be not highly effective in the regulation of metabolite biosynthesis. Additional more samples analysis and functional analysis are required to prove the activity of these TFs in regulation of metabolite biosynthesis. Additionally, transcriptomics data exhibited that most genes and TFs involved in catechins, caffeine and theanine biosynthesis are multiple copies which varied and highly correlated with the amount of metabolite enrichment in the tea leaves collected from different seasons. For example, nine genes were found to be significantly correlated with the expression of TFs and the content of secondary metabolites which played important roles in the formation of varied tea taste and nutritional function over different seasons (Fig. 7). These genes included SAMDC (CSA029628) gene in theanine biosynthesis, CHS (CSA029775), DFR (CSA003950), SCPL1A (CSA032305, CSA014045, CSA005865, CSA034015, CAS022656, CSA014844) for catechins biosynthesis. It is worth noting that SCPL1A, the largest family with 17 copies among all detected genes, can catalyze catechins to corresponding gallate catechins such as EGCG and ECG, which play crucial roles in the formation of tea astringent taste. Here we found that seven SCPL1A copies are highly associated with the expression of TFs and amounts of ECG, EGCG, and EC in different seasons (Fig. 7). Specifically, two copies (CSA014844, CSA022656) are positively correlated with ECG abundance, and negatively correlated with EC abundance. The other two genes (CSA032305, CSA005865) are only positively correlated with ECG abundance. These results indicated that different copies, expression levels of genes and environmental factors such as sunshine, temperature, and humidity in tea plants contribute directly or indirectly regulate the biosynthesis of secondary metabolites.

## Conclusions

In this study, we analyzed the transcriptome and metabolome of tea leaves collected from the seasons of Spring, Summer, and Autumn. The results elucidated that photosynthesis can regulate the expression of important genes and is correlated with TFs in metabolite biosynthesis, and consequently resulted in significant phenotype changes and varied amounts of catechins, caffeine, and theanine in the tea leaves of three seasons. Through analysis the interactions among TFs, gene expression, and metabolites abundance, nine candidate genes were recongnized highly correlated with metabolites enrichment and TFs expressions. These findings enhance our understanding of the interactions among TFs, gene expression, and metabolite enrichment and provide new insights into abundance and influencing factors of metabolites of tea in different seasons.

## Methods

### Chemical agents

HPLC-grade acetonitrile and methanol were purchased from Fisher Chemicals (NJ, USA). HPLC grade formic acid was purchased from Tedia (Lake Forest, CA, USA). Ultrapure water was produced by a Milli-Q Plus water purification system (Millipore, Bedford, MA). Commercial standards including caffeine, theanine, (+)-catechin (C), (−)-epicatechin (EC), (+)-gallocatechin (GC), (−)-epigallocatechin (EGC), (−)-catechin- 3-gallate (CG), (−)-epicatechin-3-gallate (ECG), (−)-gallocatechin-3-gallate (GCG), (−)-epigallocatechin-3-gallate (EGCG) were purchased from Sigma (Sigma-Aldrich, USA) and used to construct standard curves for quantitative analysis in the tea leaves.

### Tea tissues collection

Tea plants were grown in a garden of cheyun mountain (North: N32°11′56.03″, East: E113°46′36.95″), Xinyang, Henan Province in China with an altitude of 710 m. The local tea plants were identified as Cultivar *C. sinensis* ‘CheYunZhong’ by the committee of tea tree species of Henan province and managed by local gardeners. Six tea plants with similar size and cultural environment were selected for transcriptomic and metabolomic analysis. With the permission of the tea gardeners, the tissues (one bud and the first fresh leaf) of each plant were plucked at 8:30 AM on the 12th of April, June and September 2017 respectively. All tissues were immediately frozen in liquid nitrogen and transferred to − 80 °C for further use.

### RNA extraction, library construction, and sequencing

Collected tissues were milled in liquid nitrogen and used for RNA extraction. Total RNA was extracted using Trizol reagent (Invitrogen) separately [30, 31]. The integrity of extracted RNA was detected by Bioanalyzer 2200 (Agilent), and the RNA integrity number larger than eight is acceptable for complementary DNA (cDNA) library construction. The cDNA libraries were constructed using the TruSeq Stranded mRNA Library Prep Kit (Illumina, Inc.) according to the manufacturer’s instructions. Briefly, mRNA was purified by oligo (dT) magnetic beads and fragmented into 200–500 bp length using divalent cations at 94 °C for 5 min. The cleaved RNA fragments were used for First-strand cDNA synthesis through reverse-transcriptase and random primers. Second-strand cDNA was synthesized by cDNA and dNTP mix. The products were purified and enriched by PCR to obtain final cDNA libraries and sequenced by the HiseqX10 platform (Illumina, San Diego, CA). Finally, 150 bp paired-end reads were ultimately generated.

### Sequencing reads assembly and functional annotation

To obtain clean reads, raw data of transcriptomics were purified by removing the adapter sequences, ambiguous nucleotides (N) and low-quality reads. Clean reads were mapped to the genome sequence of *Camellia sinensis* (TPIA database, http://tpia.teaplant.org/) through HiSAT2 (Hierarchical Indexing for Spliced Alignment of Transcripts). The expression levels of all mapped reads were normalized by FPKM (fragments per kilobase of transcript per million mapped reads) method. Differentially expressed genes (DEGs) between each pair of April, June, and September were calculated respectively. The DEGs with false discovery rate (FDR) < 0.05 and ∣log 2^FC^∣ > 1 were selected as candidate genes with significant differences and for further functional analysis. These genes were aligned in Gene Ontology (GO) for functional annotation, and in the KEGG database for pathway analysis [32, 33].

### Quantitative real-time polymerase chain reaction analysis

The expression levels of DEGs were verified by a quantitative real-time polymerase chain reaction (qRT-PCR) method. Obtained cDNA products were diluted for 20-fold with nuclease-free deionized water. PCR was performed in a 20 μL reaction volume using a Bio-Rad iQ2 PCR system (Bio Rad, USA). Twenty genes were randomly selected and used to verify the expression levels. The primers were designed using the Primer Premier 5.0 program (Palo Alto, CA, USA). The *β-tubulin* gene was used as a reference due to its relative stability [34]. All primers sets used in our test were listed in Table S7. The PCR conditions were as follows: 95 °C for 1 min; 40 cycles of 95 °C for 20 s, 60 °C for 20 s, and 72 °C for 30 s. The expression levels of selected genes were analyzed using the 2^-ΔΔCt^ method [35].

### Extraction and UPLC-Q-TOF MS analysis

Eighteen tea samples collected from three seasons (six replications in each) were used for metabolomics analysis. All tissues were milled in liquid nitrogen, and lyophilized at − 80 °C for 24 h. Dry samples (~ 20 mg) were re-suspended in 360 μL cold methanol, vortexed for 2 min, and supersonic for 30 min respectively. Then, 200 μL chloroform was added into each tube and vortexed for 2 min. 400 μL ultrapure water was added in the tube, vortexed for 2 min, supersonic for 30 min. All samples were centrifuged at 4 °C and centrifuged at 14000 rpm for 10 min. The supernatant was subsequently filtrated through a 0.22 μm filter, transferred into new glass tubes, and used for later analysis.

Metabolomics analysis was conducted using a UPLC/Q–TOF–MS/MS system (Waters, USA). Quality control (QC) samples were prepared by mixing an equal amount of each sample. Metabolites in extraction were separated by a Waters BEH Shield RP C18 column (2.1 × 50 mm, 1.7 μm) at 30 °C. Two mobile phases including phase A (water with 0.1% formic acid) and phase B (acetonitrile with 0.1% formic acid) were used in the tests. The flow rate was 0.3 mL/min. The metabolites were determined by gradient elution as follows: 0–1 min, 2–5% B; 1–6 min, 5–85% B; 6–8 min, 85–100% B; 8–9 min, 100–2% B; 9–10 min, 2% B. Data were collected in the electrospray ionization (ESI) (ESI+) in full-scan mode from *m/z* 100–1000 Da. The optimized ESI parameters were as follows: capillary voltage, 3.0 kV; cone voltage, 35 V; source temperature, 100 °C; desolvation temperature, 350 °C. For accurate mass measurement, leucine enkephalin was used as the lock spray standard ([M + H] + = 556.2771 m/z) at a concentration of 200 ng/mL under a flow rate of 50 μL/min [36].

### Metabolomics data analysis

Data obtained from UPLC/Q–TOF–MS/MS were processed using Progenesis QI software (waters, version 2.1, Nonlinear Dynamics, Newcastle upon Tyne, UK) for peak alignment, normalization, signal integration, and initial compound assignments. Metabolites were identified by comparing accurate masses, MS fragmentation patterns and isotope patterns with online metabolite databases of the Human Metabolome Database (HMDB) and tea metabolome database (http://pcsb.ahau.edu.cn:8080/TCDB/f) [37]. Statistical analysis of identified compounds in tea samples was analyzed through SIMCA 14.1 software [38]. Variable importance in projection (VIP) analysis was performed to evaluate the significance of metabolites. Pairwise comparisons analysis among three groups were conducted, and the metabolites in each pair with VIP > 1 and *P* < 0.05 were considered as candidate metabolites with a significant difference.

### Quantitative analysis of catechins, caffeine, and theanine in tea tissues

Catechins, caffeine, and theanine were extracted from the tea leaves and analyzed by the HPLC equipment (Primaide, Hitachi, Japan). For the caffeine and catechins analysis, milled samples (0.05 g) were suspended in 2 mL ethanol (80% in water, v/v), supersonic at 100 w for 25 min. The solution was centrifuged at 12000 rpm for 10 min. The supernatant was obtained and filtrated through a 0.22 μm filter for further use. For the HPLC analysis, the column is LaChrom C18 (4.6 × 250 mm, 5 μm). The detection wavelength is 276 nm, the flow rate is 1 mL/min. The column temperature is 40 °C. Two mobile phases were used in the tests including phase A: water, B: methanol. The compounds were determined by gradient elution as follows: 0–40 min, 10–60% B; 40–50 min, 60–100% B.

For the theanine identification, the milled samples (0.05 g for each) were suspended in 0.75 ml water and bathed at 100 °C for 5 min. The suspension was centrifuged at 12000 rpm for 10 min. The supernatant was filtrated through a 0.22 μm filter, the obtained solution was used for the theanine analysis. For the HPLC analysis, the column is LaChrom C18 (4.6 × 250 mm, 5 μm). The detection wavelength is 210 nm, the flow rate is 1 mL/min. The column temperature is 40 °C. The mobile phases included A: acetonitrile, B: water. The compounds were determined by gradient elution as follows: 0–5 min, 10% A; 5–10 min, 10–60% A, 10–20 min, 60–10% A. Commercial compounds of catechins, caffeine, and theanine were detected through HPLC by the same method mentioned above. The quantification of each compound collected from tea tissues was performed with standard curves constructed by gradient dilutions of commercial compounds.

### Co-expression network of transcription factors, genes expression and metabolite enrichment in *C. sinensis*

To locate core Transcription Factors (TFs) in the tea plants, the co-expression network based on TFs, gene expression, and metabolite enrichment were constructed. TFs and genes involved in three biosynthetic pathways (catechins, caffeine, and theanine) were identified in transcriptomic data by homology alignment. The correlations between metabolite content and gene expression were analyzed by the Pearson correlation method. The coefficients higher than 0.9 and *P* < 0.05 were considered as significant correlations. Correlated genes highly related to metabolite content were used to test the correlations with TFs among three seasons. The correlation was constructed through Weighted Gene Co-expression Network Analysis (WGCNA) based on R (Version 3.5.1) [39]. The coefficients’ weight higher than 0.5 and *p* < 0.05 were considered as a significant correlation [40, 41] and used to construct networks through Cytoscape (version 3.6.0).

### Statistical analysis

Three independent biological replicates were tested for transcriptomic analysis, and six independent biological replicates were conducted for metabolic analysis. The PCA analysis and Heatmap of metabolomic data were conducted in metaboanalyst website (https://www.metaboanalyst.ca/MetaboAnalyst/home.xhtml) to observe intrinsic metabolite variance between tissues [42]. The Heatmap and PCA analysis of transcriptomics were performed with the R package. The correlation analysis was conducted with the R package, and the network was displayed using Cytoscape (version 3.6.0).

## Supplementary information

**Additional file 1 Table S1.** Summary of sequencing data in transcriptome.

**Additional file 2 Table S2.** The pairwise compared DEGs of tea leaves in three seasons with log2^FC^ > 1 and FDR < 0.05. AccID: ID number of genes in tea genomome database. FC: fold changes. FDR: false discovery rate

**Additional file 3 Table S3.** Pathway analysis of up- and down-regulated union genes in KEGG database with *P* < 0.05. FC: fold changes. FDR: false discovery rate

**Additional file 4 Table S4.** Functional categories of up- and down-regulated union genes in gene ontology (GO) with P < 0.05. FC: fold changes. FDR: false discovery rate

**Additional file 5 Table S5.** The correlations analysis between gene expression and amount of catechins, caffeine and theanine, respectively.

**Additional file 6 Table S6.** The correlation weight analysis of transcription factor, gene expression and amount of catechins, caffeine and theanine, respectively.

**Additional file 7 Table S7.** All primers used in qRT-PCR analysis.

## Data Availability

All data generated or analyzed during this study are included in this published article and its supplementary information files. Raw Illumina sequencing reads of tea leaves have been deposited in the NCBI Sequence Read Archive Database under accession PRJNA631799.

## Abbreviations

C: (+)-catechin
EC: (−)-epicatechin
GC: (+)-gallocatechin
EGC: (−)-epigallocatechin
CG: (−)-catechin-3-gallate
ECG: (−)-epicatechin-3-gallate
GCG: (−)-gallocatechin-3-gallate
EGCG: (−)-epigallocatechin-3-gallate
Spr: Spring
Sum: Summer
Aut: Autumn
HMDB: Human Metabolome Database
UPLC/Q–TOF–MS/MS: ultra-high-performance liquid chromatography/quadrupole time-of-flight mass spectrometry
DEGs: differentially expressed genes
GO: Gene ontology
KEGG: Kyoto encyclopedia of genes and genomes
BP: biological process
MF: molecular function
CC: cellular component
SCPL1A: type 1A serine carboxypeptidase-like acyltransferases
CHS: chalcone synthase
CHI: chalcone isomerase
ANR: anthocyanidin reductase
F3’H: flavanone 3-hydroxylase
F3’5’H: flavonoid 3′,5′-hydroxylase
DFR: dihydroflavonol 4-reductase
ANS: anthocyanidin synthase
LCR: leucoanthocyanidin reductase
5′-NT: 5′-nucleotidase
AMPD: AMP deaminase
APRT: adenine phosphoribosyltransferase
GDA: guanine deaminase
GMPS: GMP synthase
IMPDH: IMP dehydrogenase
NMT: N-methyltransferase
SAMS: S-adenosyl-L-methionine
TCS: caffeine synthase
TS: theanine synthetase
GS: glutamine synthetase
GOGAT: glutamate synthase
GDH: glutamate dehydrogenase
SAMDC: S-adenosylmethionine decarboxylase
ADC: arginine decarboxylase
