# Supplementary material for: Integrated transcriptomics and metabolomics analysis of catechins, caffeine and theanine biosynthesis in tea plant (Camellia sinensis) over the course of seasons
Source: BMC Plant Biol. 2020 Jun 29;20:294. doi: 10.1186/s12870-020-02443-y (PMC7322862; doi:10.1186/s12870-020-02443-y)
Supplement: Supplementary file 1 — Additional file 1 Table S1. Summary of sequencing data in transcriptome. [file 12870_2020_2443_MOESM1_ESM.docx]

| **Terms** | **Total Base** | **Total reads** | **GC (%)** | **Mapping reads** | **Mapping Rate** | **Unique Mapping reads** | **Unique Mapping Rate** |
| --- | --- | --- | --- | --- | --- | --- | --- |
| Apr-1 | 7157815473 | 47815126 | 45 | 39187915 | 82.00% | 36278294 | 75.90% |
| Apr-2 | 6470456863 | 43202210 | 45 | 35874077 | 83.00% | 33304576 | 77.10% |
| Apr-3 | 7338232683 | 48991096 | 45 | 40032472 | 81.70% | 37095899 | 75.70% |
| Jun-1 | 6885685175 | 45692546 | 45 | 35860801 | 78.50% | 33426173 | 73.20% |
| Jun-2 | 6484639333 | 43031904 | 45 | 33377298 | 77.60% | 31114526 | 72.30% |
| Jun-3 | 6532290014 | 43351656 | 45.5 | 33538659 | 77.40% | 31239608 | 72.10% |
| Sep-1 | 6265598751 | 41829916 | 45 | 34014546 | 81.30% | 31595028 | 75.50% |
| Sep-2 | 7012774863 | 46817138 | 44 | 37467681 | 80.00% | 34805630 | 74.30% |
| Sep-3 | 7143294634 | 47688400 | 45 | 39268383 | 82.30% | 36527745 | 76.60% |

**Table S1. Summary of sequencing data in transcriptome.**
