# Supplementary material for: Integrated transcriptomics and metabolomics analysis of catechins, caffeine and theanine biosynthesis in tea plant (Camellia sinensis) over the course of seasons
Source: BMC Plant Biol. 2020 Jun 29;20:294. doi: 10.1186/s12870-020-02443-y (PMC7322862; doi:10.1186/s12870-020-02443-y)
Supplement: Supplementary file 7 — Additional file 7 Table S7. All primers used in qRT-PCR analysis. [file 12870_2020_2443_MOESM7_ESM.docx]

| **Gene ID** | **Sequence (5’-3’)** |
| --- | --- |
| CSA023133 | CAACAAGCAGAAACCCAT  TTGGCACAAACAGAATAG |
| CSA027568 | TGCTTTGTTAAGGGTTGTG  GGTTGGGACCGTGTAGTT |
| CSA029334 | TGGGGCCGGTGTTATGAAAG  TGGTGGAAAAAGCGGAGAGA |
| CSA022828 | AGTATGATGTCCCATTGGGAAGG  TCTATTTTCGAAGGAAGTGCAGT |
| CSA034169 | GGTCTGCCGATAATTGAA  CAGCCTCTTGTGGTAGTTGA |
| CSA028401 | CAAGAAACCTCTTCGCTCTG  CGATGTCGGTGGGATAAA |
| CSA031792 | GATTGCGTGGATGGACTT  GGTGCCAGCAGTGAATAA |
| CSA006215 | GCTAACTCTAACCTCCCTG  TTATGTTGGCGTCATTGTA |
| CSA012981 | CTACCAGGGCTGATGAAA  GGAACCGTAACAGTGAGAA |
| CSA013547 | GAAAGGTGGAGGATGTCG  TTGGTGAGGCTCTTGTAG |
| CSA028406 | TGTGCTTGTCTTTGTTCCTTGT  TTCGATTTATCGAATTTTGTGG |
| CSA013563 | ACTCTACTCGGGGCTTTGGT  CTCCTGCCTGGTTTGCTGTC |
| CSA026158 | TCAAAGTAGGTGACAAAGGCGC  CAGGTTATCAGGAATGCAAAGC |
| CSA023575 | CTATGCCGATACTTGCCCTGAC  CAAACCCACCAACACCTTGATG |
| CSA017941 | CCCCTTCTCCACCACTCT  TCTTCAACTTCCACCTTCAC |
| CSA001160 | TGAAGAAAGCGATGATGAACGA  ATGCAAGGAAATATTGGCAGGT |
| CSA028450 | TTTGAATGCTGGAATATTTGGG  GTGATGTTGTGGTTGAGGAGGT |
| CSA005570 | CCCAGCCCAAGACACTAT  CTTAACCTCCCAGCCACT |
| CSA024931 | TCAACTGAGGCAGGACTT  ACTGAACCATCACAACCC |
| CSA009706 | AGAGCCAGAGGCGATGAAGG  CGGGGGCGAGAAGAGAGTAA |

**Table S7. All primers used in qRT-PCR analysis.**
